# Nonlinear phenomena in animal vocalizations: do they reflect alternative functional modes of voice control, ‘leaked’ cues to quality or condition, or both?

**DOI:** 10.1098/rstb.2024.0010

**Published:** 2025-04-03

**Authors:** Drew Rendall

**Affiliations:** ^1^ Department of Biology, University of New Brunswick, Fredericton, New Brunswick, Canada, E3B 5A3

**Keywords:** animal communication, nonlinear vocalizations, voice control, voice quality

## Abstract

Nonlinear phenomena (NLP) in animal vocalizations typically present as abrupt departures from normative controlled voicing. They occur most commonly in loud vocalizations, often in contexts of high arousal, including alarm, aggression, fear or distress, or in elaborate displays of territory or competitive ability. They therefore invite interpretation as ‘mistakes’ that evince loss of vocal control resulting from effortful, emotional ‘over-driving’ of the vocal system. However, vocal over-driving may be more flexible and purposeful, representing an alternative functional mode of voice control if NLP can benefit signallers in some contexts. The latter perspective is first elaborated with examples from non-human primates before turning to cases where NLP truly do evince loss of vocal control that may then ‘leak’ cues to signaller quality or condition. To support future frameworks to study and understand the different domains where NLP occur, a functional distinction is emphasized that turns on whether high-amplitude, effortful voicing—which inherently predisposes NLP—is at the discretion of the signaller such that the focus is on the adaptive *production* of NLP, or whether effortful voicing is effectively forced upon signallers by other dictates of the context itself, changing the focus to being the adaptive *avoidance* of NLP.

This article is part of the theme issue ‘Nonlinear phenomena in vertebrate vocalizations: mechanisms and communicative functions’.

## 1. Introduction

**Figure 1.**
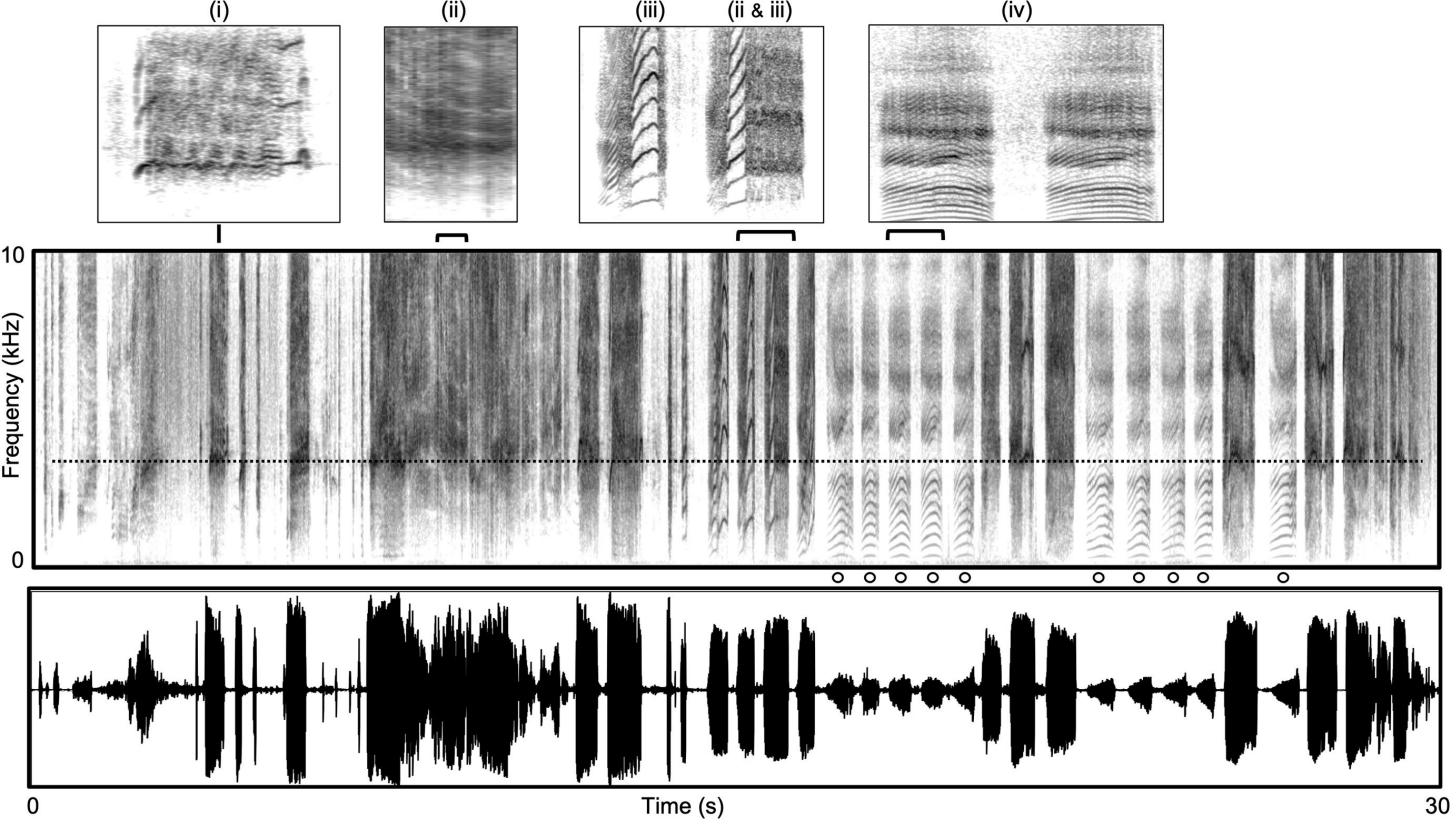
Spectrogram and waveform display of a portion of a longer weaning tantrum by an immature baboon in response to being rebuked by its mother for persistent attempts to nurse. The sequence is replete with NLP that include (i) calls with quasi-periodic high-frequency tonal elements with sidebands and a noisy overlay; (ii) calls that degrade further into broadband noise (deterministic chaos); (iii) calls that involve rapid frequency jumps; and calls with various intermediate forms and combinations. Note also that the sequence is punctuated by episodes (o) of lower-amplitude well-conditioned tonal-harmonic vocalizations (iv) that typify normative voicing. Note that there is a spectral emphasis at approximately 3.0 kHz (----) that is sustained throughout most of the high-amplitude calls in the bout. While not definitive, this may reflect a matching of some periodicity in the underlying source spectrum to a resonance (or formant) of the vocal tract, exemplifying a phenomenon, referred to as formant-matching, which is characteristic of some modes of human vocal performance as described further in §2f.

**Figure 2.**
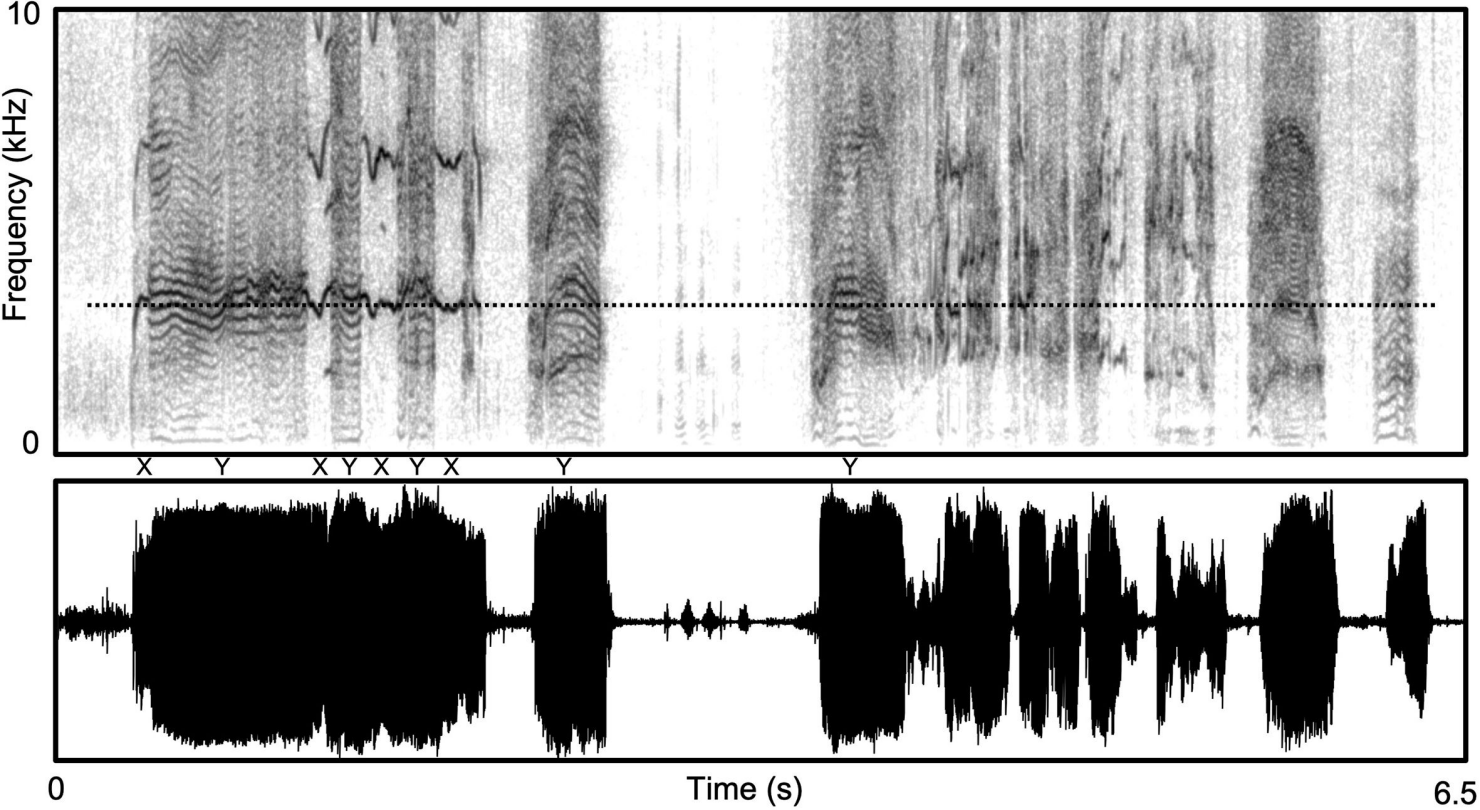
Part of a protracted bout of screams produced by the same immature baboon in figure 1 when faced with an aggressive attack by an older, larger juvenile rival. The sequence is an almost continuous stream of high-amplitude, rapidly varying calls dominated by NLP. Note in this example as well a fairly consistent spectral emphasis across the various calls in the bout, this time at approximately 3.5 kHz. In this case, the emphasis at 3.5 kHz manifests in some places (X) as a short and very high-frequency and unpredictably modulated ‘whistle-like’ tone, while in other places (Y), it takes in multiple frequency bands that could represent another example of formant-matching or possibly an alternative nonlinearity involving the intrusion of a separate low-frequency fundamental (at approx. 300 Hz) that introduces an amplitude modulation of the high-frequency carrier whistle, yielding sidebands that look like harmonics (cf. [1]).

Nonlinear phenomena (NLP) are common in the vocalizations of animals and humans. Some of the most common forms include rapid frequency jumps, subharmonics, biphonation and deterministic chaos (see figures 1 and 2 for examples). These different forms of NLP and the mechanisms underlying them are reviewed in detail elsewhere (e.g. [2–4] ‘Monkey Yodels’; [5]). Although diverse in form and in underlying production mechanics, they all share the attribute of being abrupt departures from normative voicing, the so-called modal voice register [6]. Hence, they typically manifest as transient and abrupt changes in signal structure that differ qualitatively from the well-conditioned tonal-harmonic signals that typify the stable, periodic vocal fold vibration and supralaryngeal filtering characteristic of normative voicing. As such, they involve vocal production modes (regimes) that might be considered non-normative, and it might be tempting to think of NLP as ‘mistakes’ or cases where normative voice control is ‘lost’ or there is some breakdown in ‘proper’ voice control.

Such departures from normative voicing are especially common in the vocalizations of infants and immatures [7–9], such as in the distress and hunger cries of babies and their later weaning tantrums. They are also familiar to us in humans in the voices of adolescent boys during the rapid vocal–anatomical changes associated with puberty, and they can be common among the elderly when voice quality changes again and becomes creaky, breathy or rough [6]. Together, these common manifestations of NLP might seem to reinforce the notion that they are aberrant and reflect a loss of normative voice control, and this may often be true.

However, other instances of NLP may have a specific communicative value that reflects functional (adaptive) and controllable use of non-normative vocal regimes. In this article, I first expand on this functional perspective as a deliberate counterpoint to the tradition in the field of animal communication of simply overlooking NLP or viewing them as aberrant ‘noise in the signal’. I draw on examples from a variety of animal taxa but focus on non-human primates to consider converging lines of evidence for adaptive design in and use of NLP. In this, I emphasize how NLP could, in particular, be functional to certain types of signallers in specific life stages and social contexts. From that foundation, I then flip perspectives to consider an entirely different set of contexts where the production of NLP may truly reflect a loss of vocal control, which then reveals or ‘leaks’ important cues to signaller quality or condition that could benefit listeners but possibly not signallers themselves. This juxtaposition of alternative manifestations of NLP that differentially reflect their purposeful production versus loss of control, and that differentially benefit signallers versus listeners, highlights the complex nature of voice control and some possible future research directions and challenges.

## 2. The case for functional design in and use of nonlinear phenomena

NLP are well known in the speech literature, where the focus historically has been on NLP as clinical deviations from normal healthy voicing [10–12, 13]. It is only comparatively recently that NLP have become a focus of research in animal communication (reviewed in [14,15]). In this, early reviews by Wilden *et al*. [2] and Fitch *et al*. [3] were particularly important for drawing attention to NLP in different animal taxa and promoting greater interest in categorizing their various forms and understanding the underlying mechanisms involved. These early reviews were also important in prompting a reformulation of NLP as potentially functional in nature as opposed to clinical or aberrant. The case for functional design and use of NLP now entails a variety of lines of evidence.

### (a) Nonlinear phenomena are ubiquitous across species and common within them

The first piece of evidence pointing to the potentially functional nature of NLP is simply their ubiquity within and across species. They are effectively diagnostic of the cries of distress, fear or anxiety in infants and immatures in human and non-human primates and virtually any other species whose vocalizations have been studied systematically [16,17]. They are also characteristic of the barks and screams produced by adults of many species in contexts of alarm, distress, fear or aggression (e.g. rhesus monkeys: [18], yellow-bellied marmots: [19,20], meerkats: [21], humans: [22]). They have also been documented in the whines of puppies [16,23], in the twitters of African wild dogs and the mewing of the wild marbled cat [2], the solicitation or begging calls of seal pups and immature penguins [24,25], the signature pant-hoots of chimpanzees [26] and cries produced by women during childbirth [27], to give just a few other examples.

Many of these contexts are characterized by high arousal and/or significant vocal effort. Hence, it is conceivable that the NLP involved are largely epiphenomenal, reflecting accidental loss of vocal control in such situations. However, the taxonomic ubiquity of NLP across so many different animal groups and their frequent and consistent occurrence in common contexts among them almost begs for re-interpretation in more functional terms. At some point, the sheer ubiquity of ‘mistakes’ seems more parsimoniously interpreted as a sign of potential adaptive design.

### (b) Nonlinear phenomena share some common structural and psychoacoustic properties

In fact, there are additional commonalities in many of the structural and psychoacoustic properties of NLP that likewise point to functional design. Thus, as noted, they occur commonly in contexts of high arousal, whether that be an infant experiencing pain, fear or hunger; a human mother giving birth; or any individual (immature or adult) encountering a dangerous predator, or aggressively attacking, or being attacked by a rival. And the vocalizations used in these various contexts tend to be extremely loud, even if the target listeners are quite close by, and often entail protracted sequences of highly dynamic signal structures that alternate quickly between different forms of NLP, including dramatic frequency excursions, transient episodes of subharmonics or biphonation and chaotic noise. The resulting vocalizations are thus highly salient and attention-getting. In some cases, they also have a piercing and jarring quality that can be psychoacoustically quite aversive when experienced at close range. Because NLP also often occur in protracted sequences of loud vocalizing, where the structure of successive calls changes rapidly and unpredictably, they have the additional property of being difficult to ignore, tune out or habituate to, which magnifies their effects (reviewed in [28,29]). In short, they are the kinds of sound that you are, as a nearby listener, motivated to respond to, ‘turn-off’ or avoid. (Modern car alarms are designed on the same principles.)

Some years ago, it was hypothesized that this constellation of structural and psychoacoustic properties in many of the contexts in which NLP occur was not coincidental and likely not simply an unselected by-product of the high-arousal contexts involved but rather was adaptively functional in such contexts specifically for the properties of being attention-getting and difficult to ignore and therefore well-designed to catalyse responses in others [3,28,30,31]. Thus, for example, the common cries produced by young infants in situations of distress, fear or hunger, as well as alarm calls produced by individuals of all ages when encountering a predator, are similarly not only very loud but also often chaotic in structure with noisy, aperiodic spectra. That particular combination of properties makes them highly effective in capturing and focusing the attention of other group members: in the first case, motivating carers to provide support to distressed infants; and in the second case, alerting other group members to the presence of a dangerous predator they might not yet have detected themselves ([28]; see [32] for evidence of the neurophysiological mechanisms involved).

### (c) Nonlinear phenomena share common social–functional effects

An additional social–functional commonality was also noted, namely that vocalizations of this nature are produced more frequently by individuals with limited social or physical influence over others, being either infants and immatures or adults of relatively low social standing [3,28,30]. Hence, they are less able to influence others or ‘get their way’ either by dint of force or by virtue of who they are, their dominance status and their wider social connections. It was proposed that these inherent ‘handicaps’ describe an influence inequity among the members of a group that the psychoacoustic properties of certain kinds of vocalizations might help to rebalance [28–30]. In other words, while physically or socially impotent to exert their will on others, immatures and adults of lower social standing are not entirely helpless—they have some other tools at their disposal to help them overcome being routinely dominated or ignored.

For example, an older infant attempting to nurse from a resistant mother who has begun the weaning process, or that is trying to thwart the aggressive advances of an older, larger or higher-ranking rival, might succeed to a degree in both contexts using similarly structured vocalizations. In the first instance, a loud, hypervariable stream of aversive vocalizations might wear down a mother who is reluctant to acquiesce to the infant’s continuing demands to nurse but is also tired of the ‘harangue’. In the second instance, a rival might be ‘persuaded’ to relent in the face of a similar barrage of aversive vocalizations at close range, which effectively tests its motivation to persist in what was, in any case, possibly fairly gratuitous harassment of the youngster to begin with [29].

Figures 1 and 2 illustrate a portion of a protracted weaning tantrum and then a bout of agonistic screaming, both from the very same immature baboon. In the first instance, the vocalizations are directed at its mother; in the second at an older, larger peer. In both cases, the calls are extremely loud—many times louder than they need to be to reach their targets who are right in front of them—with multiple forms of NLP and aversive qualities that are difficult to ignore or tune out. Why would the vocalizations be so similar in these two very different contexts, one involving the infant’s own mother, who is, after all, its primary supporter and carer, and the other involving a competitive rival? Perhaps it is because in both situations the infant is facing a resistant, uncooperative or outright aggressive target who is physically and socially more potent and thus typically able to dominate or control the infant. For the infant then, one possible way to regain some degree of influence in both situations is by producing a stream of otherwise unnecessarily loud vocalizations with rapidly varying, unpredictable and perceptually aversive properties that challenge the will of both mother and rival. Of course, this vocal strategy might not be without risks if the aversive nature of the infants’ protests actually provoke escalated resistance or attack by the mother or rival rather than their acquiescence, a possibility returned to again below.

While originally proposed for specific classes of vocalizations among non-human primates, these arguments likely have wider applicability. Indeed, similar arguments have now been made for the structural design and function of similar types of vocalization in a host of other taxa ranging from humans, red deer and dogs to marmots and meerkats, where NLP have been reported specifically to increase call salience and preclude habituation [19,21–23,33].

### (d) Purposeful, controllable production of nonlinear phenomena

Notwithstanding all of the aforegoing, it could be that many of the NLP observed across species are largely incidental by-products of vocal ‘over-driving’ in contexts of high arousal—in other words, they do really represent a ‘loss of control’ induced by high states of arousal that push the vocal production system out of control. That loss of control might still be functional in the ways already discussed, but the NLP could simply result from, and thus index, the signaller’s highly aroused state (cf. [34]). Alternatively, it is possible that, despite the emotionally charged nature of the situations, signallers are nevertheless able to exert some control over the production of NLP.

On this latter point, the weaning tantrum illustrated in figure 1 is potentially illuminating. The calls produced in this protracted bout of vocalizing are replete with NLP that take various forms, including rapid frequency jumps, highly dynamic quasi-periodic structures with rapid frequency modulations, deterministic chaos and almost everything in between. However, the bout also entails short episodes of well-conditioned, highly periodic and harmonically rich calls, which are signal structures that typify normative voicing. Among primates, calls of this latter form are typically produced in relatively relaxed and affiliative social contexts, such as grooming or as contact calls during group travel. Note also from the figure that these latter calls are also much lower amplitude compared with those in which NLP appear. Hence, true to theory, the well-conditioned periodic signals are associated with relatively low-amplitude controlled voicing, while NLP appear in the high-amplitude calls, perhaps reflecting now a loss of control as a result of over-driving the vocal system. But is this over-driving simply a by-product of high arousal, resulting from and therefore only indexing each youngster’s extremely agitated state? Or could the vocal over-driving be, in some sense, purposeful?

To argue the former, we would have to assume that the youngster is pinballing back and forth between states of extreme arousal and agitation to virtual calm, cycling emotionally back and forth several times across the course of this short bout of calling. Is it instead possible—and even more parsimonious—that the youngster is, in fact, more purposefully toggling back and forth not emotionally but between relatively controlled normative voicing and deliberately over-driving the vocal system so as to induce NLP abruptly and unpredictably over the course of the calling bout? That is, despite the emotionally laden situation, the youngster nevertheless retains some degree of control over both forms of voicing and can therefore flexibly adjust the ‘settings’ to maintain normative vocal control but then also purposefully over-drive the system to produce a variety of NLP as well, toggling between the different voicing modes as the situation unfolds.

Assuming this latter interpretation is plausible, a question then becomes why the youngster also includes low-amplitude harmonic calls in protracted bouts of calling like this. That is, if, as already argued, there is functional value to producing a hyper-variable stream of high-amplitude calls with diverse NLP to exert some influence over and preclude habituation by resistant or aggressive targets, what further value might there be in punctuating this stream of aversive calls with some that are lower-amplitude, tonal-harmonic and far less aversive in nature? The possibility that suggests itself is that this too contributes to the unpredictable nature of the sequence that makes it challenging to ignore: with variable numbers of successive low- versus high-amplitude calls, the listener simply does not know when the next break from, or the next swing to, the more aversive high-amplitude over-driving will occur. And that adds to the psycho-emotionally frustrating nature of the experience for the listener. Further, and not mutually exclusively, it may be that punctuating the sequence of aversive high-amplitude calls that include NLP with episodes of less aversive, quieter forms of normative voicing associated with positive, affiliative contexts helps to modulate the aversive effects of the former call types to induce a more nurturant response from the mother, thereby avoiding the possibility noted earlier that protracted sequences of aversive vocalizations replete with NLP might, counter-productively, induce *more*ORE rather than less resistance from target listeners. This possibility is a kind of parallel to the beloved pet Fido’s alternation between high-frequency (grating) whines and softer whimpers with drooped ears, lowered head and plaintive stare, which combination is designed to get and hold your attention but then to secure your succour in the form of a tasty treat or at least a pat on the head. The juxtaposition of ‘attention-getting-yet-endearing’ is particularly hard to resist.

These last points are a bit speculative but are arguably worth pursuing with a close examination of similar extended vocal sequences in other species and contexts to provide some broader examination of how common this sort of rapid and potentially flexible juxtaposition of normative voicing and NLP is. Through such comparisons, we might better establish the degree to which NLP are under flexible, purposeful control, further buttressing the case for functional adaptive design, or indeed are more tightly leashed to emotional states and thereby possibly largely epiphenomenal or incidental to those, even if no less functional as a result.

### (e) Other special adaptations—supplemental vocal membranes

A further piece of evidence for functional adaptive design and use of NLP comes from an additional supplemental structure that has been found on the medial edge of the vocal folds in a variety of mammal species, including several species of primates, some rodents and also echo-locating bats [35–42]. It has been variously referred to as a vocal membrane, lip or flap distinct from the main body of the vocal folds, but across these diverse taxa, it is commonly understood to represent an additional independent oscillator capable of alternative vibratory modes not possible with the full body of the vocal folds. For example, because the membrane is much smaller than the main body of the vocal folds, it naturally oscillates at much higher frequencies and with much greater efficiency (i.e. lower driving pressure than would otherwise be required). Hence, it appears particularly designed for high-amplitude, high-frequency phonation with relatively low effort [39] and is held to be responsible for some of the common and especially high-frequency tones and broadband frequency sweeps characteristic of the ultrasonic chirps of echo-locating bats, the broadband ‘song’ of some rodents and the high-frequency contact calls of marmoset monkeys and squeals of Sykes’ monkeys [35–37,40,41]. It is likely also involved in many of the high-amplitude NLP manifest in other primate vocalizations, including some of those previously illustrated in the weaning tantrums and agonistic screams in figures 1 and 2.

Ultimately, this vocal membrane appears to be an adaptive vocal design feature, supplementing the phonatory capacities of the canonical vocal folds and yielding additional functional modes of voicing that may specifically include efficient induction of NLP [3,30]. Notably, the vocal membrane characteristic of many primate species has been lost in humans [43]. This is an illuminating vocal anatomical change likely reflecting the evolutionary emergence of language in humans, where the focus for strategic influence over others shifted qualitatively from the regular use of high-amplitude vocalizing involving NLP for their *direct* perceptual influence on receivers, as is common among non-human primates, to a more *indirect* form of cognitively mediated influence involving instead the transmission of complex (semantic) information carried through the normative voicing that is characteristic of contemporary human speech (cf. [44]).

Taken together, the picture that emerges is that diverse forms of NLP that occur in loud vocalizations produced in various species and contexts may result from high-amplitude, effortful vocalizing that over-drives the vocal apparatus but not always as mistakes that evince a loss of control. Instead, they may often represent an alternative functional mode of voicing, possibly flexibly and purposefully controlled, supporting a number of adaptive purposes, including securing attention, support or investment or exerting social influence in other ways.

### (f) Human musical voice—deliberate performative registers

Further support for this expanding functional perspective on NLP comes from research on human vocal performers. Neubauer *et al*. [45] provide an accessible review of several forms of NLP common among non-classical vocalists. They include phenomena very similar to those that typify the primate vocalizations reviewed here including rapid bifurcations, subharmonics and deterministic chaos (see figs 1–6 in [45]). For vocal artists, these are found to arise from purposeful manipulation of subglottal air pressure and/or controlled desynchronization of left and right vocal folds.

**Figure 3.**
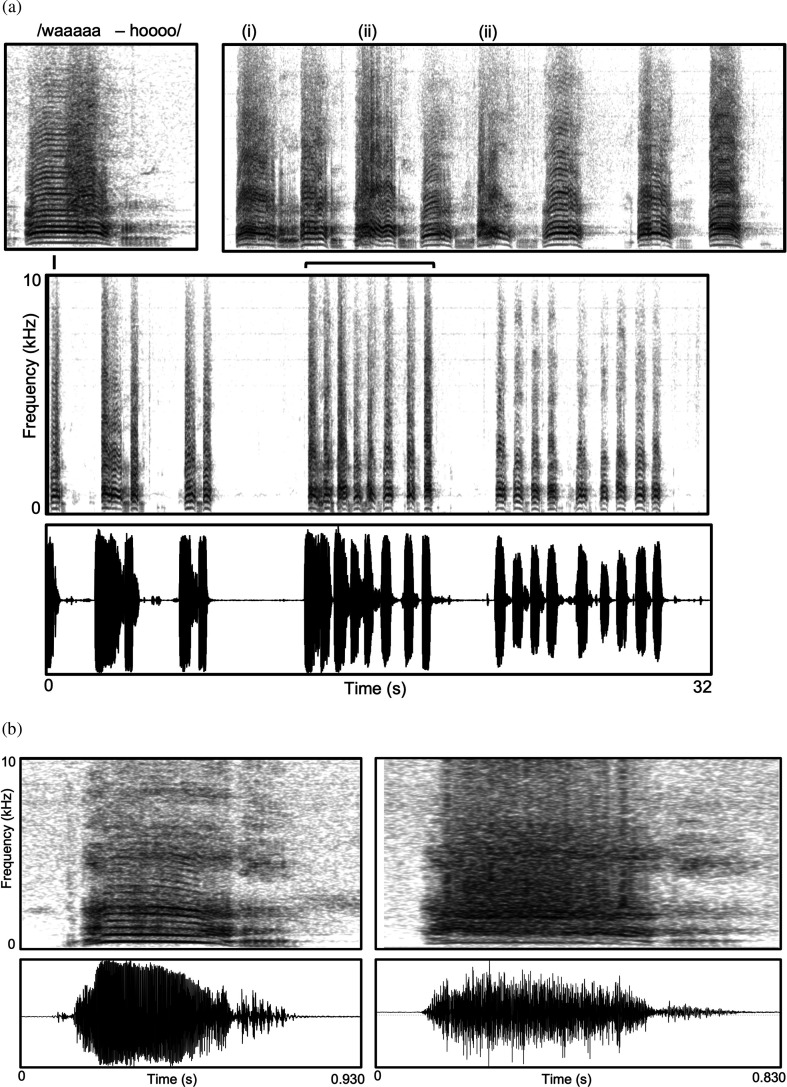
‘Wahoo’ vocalizations of adult male baboons produced as part of elaborate physical displays of competitive ability. These are two-part calls where the first (waaaa) part of the call is egressive, produced on forceful exhalation, and the second shorter section of the call (/hoo/) is ingressive, produced as part of effortful inhalation to replenish lung volume. Despite being the loudest calls in the species’ repertoire, most of the calls in the sequence in (a) retain a well-conditioned harmonic structure reflecting stable, periodic vocal fold vibration typically associated with much lower amplitude normative voicing. Only in some calls does this periodic structure break down with the appearance of subharmonics (i) or aperiodic, chaotic noise (ii). This difference is shown more clearly in (b) which illustrates two vocalizations from the same adult male separated in time by approximately 30 s. The first of the two calls is relatively harmonic with some breakdown in that structure only at the very beginning and again at the end of the /wa/ portion of the call, whereas in the second call, the harmonic structure has deteriorated into deterministic chaos throughout, which gives it a much rougher and harsher perceptual quality. The fact that most calls preserve a clear harmonic structure, with conspicuous NLP appearing in only some of them, is consistent with the proposal that selection in this context favours the preservation of stable voice control and avoidance of NLP in the face of significant challenges to that as part of the demonstration of male quality, stamina and condition.

Another phenomenon they describe is referred to as a ‘glottal whistle’, which is a very high-frequency pure-tone sound (very much like a mechanical whistle) that they attribute to vortices arising either aerodynamically from air forced through partially adducted vocal folds or some other constriction of the supraglottal airway, or via vortex-induced vibration of the vocal folds.

A further phenomenon they review is termed ‘formant-matching’, which is a form of source-filter coupling where a resonance (or formant) of the vocal tract is matched to a particular frequency in the underlying source spectrum. This correspondence between source and filter has the effect of emphasizing that particular frequency relative to others in the spectrum but potentially also introducing additional periodic vibrations in sympathy with the underlying source that manifest in the spectrum as subharmonics or sidebands.

Notably, both of these latter phenomena—glottal whistles and formant-matching—appear very similar to some of the calls illustrated previously for non-human primates (see legends of figures 1 and 2 for further descriptions and compare with figs 5–7 in [45]).

Borch *et al.* [46] describe an additional form of NLP characteristic of the vocal style of some rock and heavy metal artists, which is known in that industry as the ‘dist’ tone and entails a high-amplitude rough or harsh voice quality. It is used as the baseline singing mode for some artists or as an occasional departure from a smoother, more melodic voice for others. They use as examples of this voice quality the signature voice styles of AC/DC (i.e. original lead singer Bon Scott and subsequently Brian Johnson, who emulated Scott’s iconic voice) and Whitesnake (David Coverdale), who will be familiar to some. Arguably, similar ‘dist’ tones and related rough voice qualities characterize many other popular vocal artists, such as Bruce Springsteen (think in particular ‘Born in the USA’), Bob Seger (‘Old Time Rock-and-Roll’), James Brown (‘Whaaaaaaa, I feel good’) and Joe Cocker (just about any song, but classically in ‘A Little Help From My Friends’), to name a few.

Borch *et al.* [46] show that the ‘dist’ tone arises from vibrations of the supraglottal mucosa, which is not normally engaged in routine human voicing but arises from particularly high-amplitude voicing using very high subglottal air pressures. This effortful over-driving introduces a novel vibratory mode of the supraglottal mucosa that can be sympathetic with the periodicity of vocal fold vibrations, creating subharmonics at one or more integer fractions of the fundamental frequency, or can be aperiodic, creating a noisy overlay. Here again, manifestations of the ‘dist’ tone seem very similar to some of the NLP observed in the harsh calls illustrated here for non-human primates (compare figures 2 and 3 with fig. 3 in [46]). Notably, in humans, the loud, effortful voicing of career vocalists who regularly employ ‘dist’ tones and other high-amplitude voicing registers (e.g. belting) associated with either classical (operatic) or non-classical genres (rock-and-roll, metal) may be damaging to long-term voice health [46–48]. It is tempting to link this risk, in whole or in part, to the loss in humans of the vocal membrane, which, as noted earlier, specifically facilitates high-amplitude voicing and NLP with less effort, and presumably then less risk to vocal damage, in many animal taxa.

Whatever the possible health risks, the NLP entailed in ‘dist’ tones used by popular vocalists can contribute to listener enjoyment for some listeners (devoted fans), although they may also account for the opposite impression for some others (!) and for reasons reviewed earlier. However, the upshot for present purposes is that, for vocal artists, NLP can arise as very deliberate, controlled and reliable patterns of voicing, representing an alternative vocal register used for specific musical effects (see [49] for broader adoption and simulation of NLP in traditional and contemporary music). Of course, this fact does not mean the same is necessarily true of the examples reviewed for non-human primates, even if the NLP involved appear structurally very similar between the two groups. After all, for vocal artists, the voice is effectively an accessory musical instrument with which they have considerable expertise. Therefore, it may or may not be fair to compare vocal artists and their production of NLP to the routine use of voice and production of NLP by non-human primates. Notwithstanding that, to say that either group has deliberate control over the production of NLP would not mean there are not also times when they actually lose control. Even experts make mistakes.

## 3. An analogy: automobile driving

Consider an analogy, that of driving a car, which is something familiar to most of us. Just as we are experienced with the basic control mechanisms of the voice (lung pressure, glottal tension, oro-pharyngeal cavity articulations), we are experienced with the use of the basic control mechanisms of the car: steering wheel, accelerator and brake. Routine (and safe) driving generally involves slow steady acceleration, slow steady braking and gradual turning manoeuvres. At times, however, we lose control, for example when slippery road conditions induce uncontrolled skids or when muddy ground or deep gravel ‘take control’ of the steering wheel. These are effectively cases of NLP in the domain of automobile driving: cases where some incremental change, either in our braking or steering or in the external road conditions, induces a sudden qualitative (and often terrifying) change in the behaviour of the car. These are also examples of nonlinearities in driving where we have patently ‘lost control’.

However, there are other times when we deliberately induce such states, for example deliberately skidding to avoid hitting another vehicle or object, or (dare I mention) deliberately ‘peeling out’ or spinning doughnuts to impress friends or bystanders. Of course, some people are more skilled in inducing such controlled nonlinearities in automobile driving. Professional race car drivers, for example, are among the most skilled drivers and arguably can induce and also control these and likely many other nonlinearities in a car’s behaviour. They might be considered the automobile-driving equivalent of vocal artists who have substantially more experience than the rest of us, which includes very deliberate and organized practice controlling a car’s behaviour and inducing and managing resulting nonlinearities; in much the same way, vocal artists have deliberate practice and resulting special skills in controlling their voice and inducing functional nonlinearities.

However, very importantly, even the professional race car driver can, at times, lose control. Road or track conditions change unexpectedly, tyre conditions or brake pads deteriorate. And unintentional and uncontrollable skids or other abrupt departures can occur with dangerous consequences. Vocal artists likely also sometimes lose control of their voice, perhaps because they are a bit more dehydrated than usual, they have a touch of the flu, the venue humidity is extreme, etc., or perhaps simply because they push too hard to hit a high note or to excite the crowd. The result may be some significant (possibly cringeworthy) departure from normative voicing or controlled NLP.

Ditto with non-human primates and other animals. Even allowing that they might have significant vocal control that facilitates adaptive and functional use of NLP, it is very likely that they sometimes also ‘lose control’. In other words, even if the evolved use of voice in animals is best likened to that of expert users—the vocal artist or race car driver—we should not overplay this functional perspective on NLP. Even experts make mistakes.

## 4. Nonlinear phenomena as mistakes that leak cues to quality or condition

So, in a flip of perspective, it is important to return to the possibility of inadvertent or mistaken production of NLP. As previous reviews of NLP in animal vocalizations note, the vocal tract is composed of a number of different coupled oscillators and resonators, including the vocal folds proper, the false vocal folds (ventricular folds), the arytenoid cartilages, the epiglottis, the velum, the supralaryngeal airways, and for some species also additional vocal membranes, supplemental air sacs or enlarged hyoids [2,3]. Collectively, these constitute a fairly complex apparatus with multiple degrees of freedom, and whose coordinated integration might then seem positively prone to mistakes [3].

With this flip in perspective, it may thus be that avoiding the production of NLP arises as another target of selection, at least in some contexts, such that it becomes specifically the *absence* of NLP that is functional and where the occurrence of NLP then evinces some inadvertent loss of control that reveals or ‘leaks’ cues to signaller quality or condition that are of value to listeners but not to the signallers themselves [3,26].

This sort of scenario seems most likely to characterize contexts where signallers are not so free to flexibly over-drive the vocal system as suits their interests alone but where other dictates of the context itself demand such over-driving. In other words, contexts where the social–behavioural context itself demands sustained high-amplitude or otherwise effortful voicing that challenges signallers’ ability to maintain stable voice control and where doing so effectively advertises superior quality.

One such context would be the loud calls and elaborate territorial displays of some primates (and many other animal taxa), among the most impressive being those of the gibbon and siamang, where monogamous pairs produce extremely loud and synchronized song duets to advertise their territory-holding status to distant neighbours and individual floaters. The calls have to be extremely loud to carry long distances across the tropical forest, and additional details of the song and its synchrony are hypothesized to advertise the strength and quality of the pair bond [50,51]. Notably, these loud duets are often also accompanied by dramatic locomotor acrobatics, as male and female brachiate through the treetops in ricochetal fashion, abruptly changing speed and direction. The high amplitude nature of the song, by itself, predisposes transient departures from modal voicing, but that basic challenge is significantly exacerbated by the additional physical and aerobic demands: the biomechanical demands of high-speed brachiation exert extreme and rapidly changing forces on the bony and muscular anatomy of the upper body and torso, which are simultaneously responsible for maintaining stable lung pressure to sustain controlled vocalizing; and the additional aerobic demands of the display compound that challenge by necessitating exaggerated breathing rhythms that further handicap the ability to regulate lung pressure and glottal airflows to maintain stable vocal fold vibration.

Loud vocal displays of territory are common to a variety of other primate species (e.g. chimpanzees, orangutans, indri, mangabeys, black-and-white colobus, howler monkeys, titi monkeys). Many of these species have additional specialized adaptations of vocal anatomy (e.g. enlarged jaw or hyoid, accessory throat sacs) that serve to amplify, resonate or otherwise augment the territorial calls, which fact underscores these vocal displays as a particular target of selection. It, therefore, seems plausible that, although not yet widely considered or studied, an additional target of selection on such vocalizations might well be on maintaining stable voicing and avoiding NLP in the face of extreme challenges to that.

Loud vocal displays occur in other social contexts that throw down similar challenges, such as in the ritualized mating displays or displays of dominance or competitive ability common in some primates and many other animal taxa. A good example in primates is the loud ‘wahoo’ vocalizations of adult male baboons. As illustrated in figure 3, they can include NLP. The calls are produced as part of protracted bouts of aggressive chasing and posturing that occur when there is some challenge to the male hierarchy from other males within or outside the group or when one or more females within the group is in oestrus and males are aggressively competing for mating access. In these situations, adult males will engage in extended chases, racing at high speed around the other group members (who are often shrieking and darting out of the way), sometimes leaping up into the low branches of a tree, bounding through it and conspicuously shaking its branches, plunging to the ground and resuming the chase there, all the while producing a continuous stream of extremely loud wahoos [52,53]. Often, it is a single pair of males chasing each other, but there can sometimes be multiple males involved, contributing to the general pandemonium. It is a highly charged scenario aimed at demonstrating a male’s competitive ability and condition to intimidate rivals and impress others (e.g. females).

Although such chases sometimes escalate to actual fighting, which can be quite dangerous [53], a curious feature of many chases is that, for all the implied aggression, males frequently fail to catch one another. Indeed, one male will chase another while vocalizing loudly, but as soon as he draws close to his rival, his pace will slow a bit, ensuring that he never quite catches him. And then the chasing roles may suddenly flip, as the male being chased pivots on his pursuer and becomes the chaser. And the chasing and vocalizing continue. And now the new chaser fails to catch the original one, whereupon the roles may reverse yet again. And this cycle can continue for many minutes. Males are visibly exhausted by the end, and that seems to be the point [52,53]. The fact that males appear not to actually want to physically tangle with their rivals betrays these encounters as ritualized displays specifically for purposes of advertising one’s own and evaluating a rival’s stamina, physical condition and competitive ability without risking injury, if at all possible.

Here then is another elaborate display context that seems ripe as a target of selection on voice control, where what is favoured is specifically the ability to sustain high-amplitude stable voicing and avoid inadvertent nonlinearities in the face of physically exhausting activity that effectively handicaps that. In effect, the voice becomes a very clear barometer of quality, providing listeners with a veridical (unfakable) readout of signaller stamina and overall condition. It is, quite simply, impossible to conceal being out of breath. Hence, those in top condition can sustain the displays with little or no decrement in voice quality or calling rate, while those in worse condition will reveal that either by leaking NLP that betray transient loss of voice control or perhaps also by reducing the rate or amplitude of vocalizing to avoid that eventuality. (Any person who has had the experience of trying to sustain a conversation while running with a more fit partner will relate immediately.)

These ideas have not been systematically tested, but Fischer *et al*. [52] did find that other canonical features of baboon wahoos (fundamental frequency and duration of the ‘hoo’ portion) differed with the duration of calling bouts, consistent with effects of exhaustion; while Fischer *et al*. [54] noted some nonlinearities in the calls but did not pursue them as potential indices of quality, stamina or exhaustion. Riede *et al*. [26] noted a host of different NLP in the signature loud calls (pant-hoots) of chimpanzees and specifically proposed but did not test that they might be leaked rather than deliberate and thereby serve as inadvertent cues to male competitive ability or short-term health and condition. De Gregorio *et al.* [55] implicate vocal fatigue as one possible factor relevant to the prevalence of NLP in the loud ‘songs’ of Indri.

Altogether then, it appears a particular productive focus of future research could be on sustained loud display vocalizations—particularly those that accompany other physically and aerobically taxing activity—as a special crucible for selection on (the avoidance of) NLP as manifestations of inadvertent loss of voice control revealing cues to (poorer) signaller quality or condition.

## 5. Conclusions

NLP are a growing focus of research in animal communication, reversing a historical trend to overlook them or treat them as ‘noise in the signal’. With additional research attention, NLP will likely be found in a very diverse array of species, vocalizations and contexts, including relatively relaxed situations involving otherwise normative voicing where they might then reflect some form of vocal pathology or idiosyncrasy of individual voicing [3]. Ultimately, the many possible manifestations of NLP across diverse species and contexts may reflect many different functions (cf. [1,56]) that therefore resist simple typologies, easy synthesis or unitary explanations.

However, to date, and across a diverse range of animal taxa, a singular commonality noted here and previously is that NLP seem most likely to occur and be most conspicuous in extremely loud or otherwise effortful vocalizations, often in contexts of high arousal, sometimes accompanied by elaborate physical displays. Hence, I have focused on manifestations of NLP of this sort and have emphasized that these commonalities are not coincidental because they describe precisely the sort of conditions that challenge the ability to maintain stable voice control. They are all contexts where the vocal production system is effectively ‘over-driven’.

To help advance the expanding research focus on NLP and foster the development of productive future frameworks for tackling their study in a systematic way, I have delineated two broad and functionally distinct domains for such vocal over-driving. One domain involves cases where I have proposed that vocal over-driving is largely the signaller’s prerogative and hence may be purposefully and flexibly used specifically to induce NLP for the adaptive influence this provides signallers in certain contexts or life stages. This sort of scenario is illustrated by the examples of alarm calls, distress cries and submissive shrieks and screams of many species.

The second broad domain concerns cases where I have proposed that vocal over-driving is not the signaller’s prerogative alone but rather is prescribed by the context itself. This scenario is illustrated by examples of loud territorial displays or displays of dominance, condition or competitive ability where loud or effortful vocalizing is required by, for example, the need for long-distance signal transmission and/or arises as a result of obligatory accompanying display behaviours that are physically and aerobically taxing. These are contexts where quality or condition may be signalled specifically by the ability to now sustain stable voicing and avoid NLP in the face of significant and unavoidable challenges to that, and where the NLP that do occur are therefore more likely to reflect mistakes that leak information that benefits others but not the signallers themselves.

In both domains then, NLP are envisioned as a functional target of selection, the difference between them being that in one, the focus for signallers is on the functional *production* of NLP, while in the other, it is on functional *avoidance* of them. Discriminating between these two functional domains in any specific case where NLP occur may depend on details specific to the particular species or context. However, a possible clue as to which functional domain is entailed may lie in the extent to which NLP are a characteristic feature of vocalizations produced by most or all individuals in the same circumstances and/or NLP occur in a substantial proportion of the vocalizations produced in a given sample of calling by a particular individual (likely reflecting functional production of NLP to exert influence); or whether NLP appear only occasionally in some calls within protracted bouts of calling by a particular individual or in the calls of only some individuals (likely reflecting functional avoidance of NLP as leaked cues to quality or condition). These are imminently testable proposals and predictions for future research.

## Data Availability

This article has no additional data.
